# Clinical outcomes after treatment of quadriceps tendon ruptures show equal results independent of suture anchor or transosseus repair technique used – A pilot study

**DOI:** 10.1371/journal.pone.0194376

**Published:** 2018-03-19

**Authors:** Stefan Plesser, Mohammad Keilani, Gyoergy Vekszler, Timothy Hasenoehrl, Stefano Palma, Martin Reschl, Richard Crevenna, Stefan Hajdu, Harald Kurt Widhalm

**Affiliations:** 1 Department of Trauma Surgery, Medical University of Vienna, Vienna, Austria; 2 Department of Physical Medicine, Rehabilitation and Occupational Medicine, Medical University of Vienna, Vienna, Austria; 3 Department of Trauma Surgery and Sports Traumatology, Danube Hospital, Vienna, Austria; Mayo Clinic Minnesota, UNITED STATES

## Abstract

Biomechanical studies have shown the use of suture anchors (SA) to be superior to the traditional transosseous sutures (TS) in the repair of quadriceps tendon rupture (QTR). This study aimed to analyze and compare the functional outcomes of patients treated for quadriceps tendon ruptures using suture anchors or transosseous sutures. Patients having undergone suture anchor repair or transosseous suture repair for quadriceps tendon rupture between 2010 and 2015 at one of the two participating hospitals were included. Patients from site A underwent TS repair (TS group) while patients from site B underwent SA repair (SA group). Exclusion criteria included previous or concomitant injuries of the involved knee, penetrating injuries and pre-existing neurological conditions. Clinical outcome was assessed by subjective scores (Lysholm and Tegner Scores, International Knee Documentation Committee (IKDC) Score, Visual Analog Scale (VAS) for pain), quadriceps isokinetic strength testing, Insall-Salvati Index (ISI), and physical examination. Non-parametrical statistical analysis was conducted using the Mann-Whitney *U* test. Twenty-seven patients were included in the study of which 17 patients (63%) were available for follow-up (SA group: 9, TS group: 8). All patients were male with a mean age of 62.7 (SD: 8.8) and 57.9 (SD: 12.7) years for the SA group and TS group, respectively. The groups did not differ in terms of demographic characteristics. No clinically significant differences were identified between the two groups. There were no re-ruptures in either group. Treatment of quadriceps tendon rupture using suture anchors provides a clinically valid alternative treatment to the gold-standard transosseous suture repair.

## Introduction

Quadriceps tendon rupture (QTR) is a rare injury (1.37/100 000 patients per year) commonly affecting men (male to female ratio 4.2/1), especially between the age of 50 and 60. [1,2] The use of transosseous sutures (TS) for ruptures at or near the osseotendinous junction is an established procedure which has been used for decades, thus making it the gold-standard in treating such ruptures. [3,4,5] Suture anchor (SA) repair is a fairly new procedure where two to three suture anchors are screwed into the proximal pole of the patella.

To date, few cases of SA repair for ruptured quadriceps tendons have been published, all showing comparable outcomes to TS repair. [5,6,7,8,9] Recent cadaveric studies have attested the biomechanical superiority of suture anchor repair. [10,11] Several advantages of SA repair over TS repair have been suggested in literature including biomechanical superiority allowing for early functional rehabilitation. This can result in a more rapid recovery and better functional outcomes. [5] Furthermore, avoiding dissection of the apex of the patella reduces operative time, avoids trauma to the patellar tendon, and avoids the placement of non-absorbable knots at the apex of the patella. [7,12] Certain literature also suggests a reduced risk for patellar fractures. [13] Although SA repair possesses many proposed benefits, such as less invasive approach and shorter hospital stay, TS repair is more cost-effective. [7]

The main purpose of this retrospective cohort study was to compare the functional outcomes of patients who were treated for quadriceps tendon ruptures with either transosseous suture repair or suture anchor repair. We hypothesised that suture anchor repair would show similar outcomes to the gold-standard transosseous suture repair.

## Materials and methods

This study was approved by the Ethics Committee of the Medical University of Vienna (EK-Nr 1398/2015). Written informed consent was obtained from all the patients prior to enrolment. Patients who had been operated on a ruptured quadriceps tendon between 2010–2015 with either transosseous sutures (TS group) or suture anchors (SA group) in one of the two involved trauma centres were included. They were examined at a mean follow-up of 46 months (SA group) and 29 months (TS group). Patients from site A had undergone TS repair (TS group) and followed a conservative standard rehabilitation protocol. Patients from site B had undergone SA repair (SA group) and followed a more aggressive rehabilitation protocol. Patients were excluded if they had previous or concomitant injuries involving the affected knee, penetrating injuries, or pre-existing neurological conditions. All participating patients were contacted by mail or by phone and invited to participate in follow-up examinations.

### Surgical techniques

QTR was diagnosed by clinical examination and confirmed by ultrasound. Radiographs were performed to rule out patellar fractures and evaluate the height of the patella in the lateral view. Surgery was carried out through a midline incision, varying in length between the two operative techniques. Tourniquet use was at the surgeon’s discretion. Retinacular tears were repaired with multiple interrupted sutures using absorbable Vicryl sutures. Prior to skin closure, the leg’s range of motion (ROM) was tested to ensure the strength of the repair, ensuring not to exceed 90°. Following postoperative immobilization, patients were referred to non-specific physical therapy including quadriceps strengthening exercises and gait education.

#### TS group

**Fig 1:**
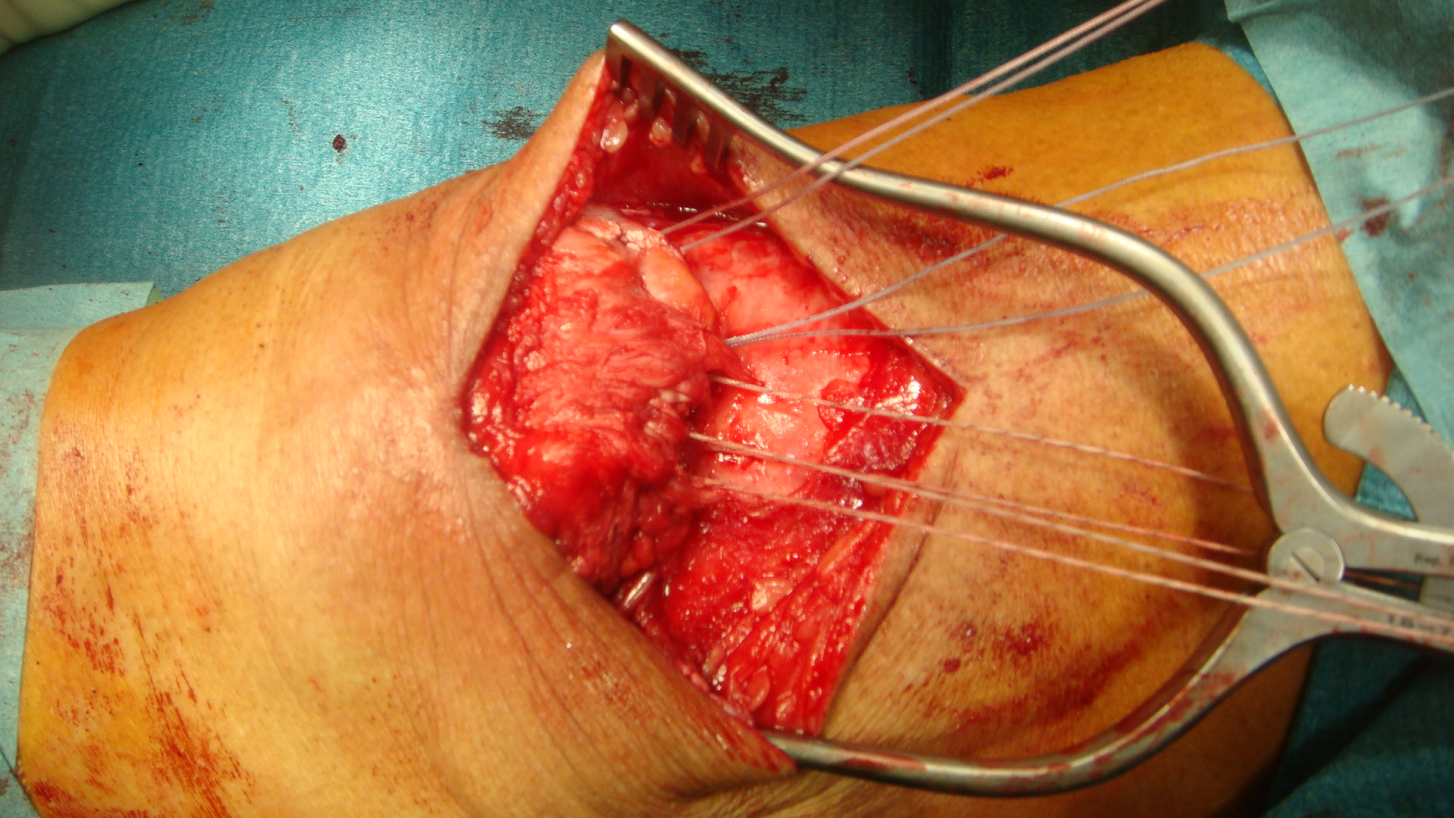
Transosseous sutures passed through patella.

TS repair included drilling three to four longitudinal tunnels through the patella and suturing the tendon using either #5 Ethibond, polydioxane (PDS) or FiberWire. (Fig 1) Suture patterns used included the Mason-Allen technique, Krackow technique and Kessler-Kirchmayr technique. Patients were usually immobilized with a cylinder cast for 6–8 weeks postoperatively, depending on the surgeon's preference. Partial weight bearing was encouraged two days after surgery.

#### SA group

**Fig 2:**
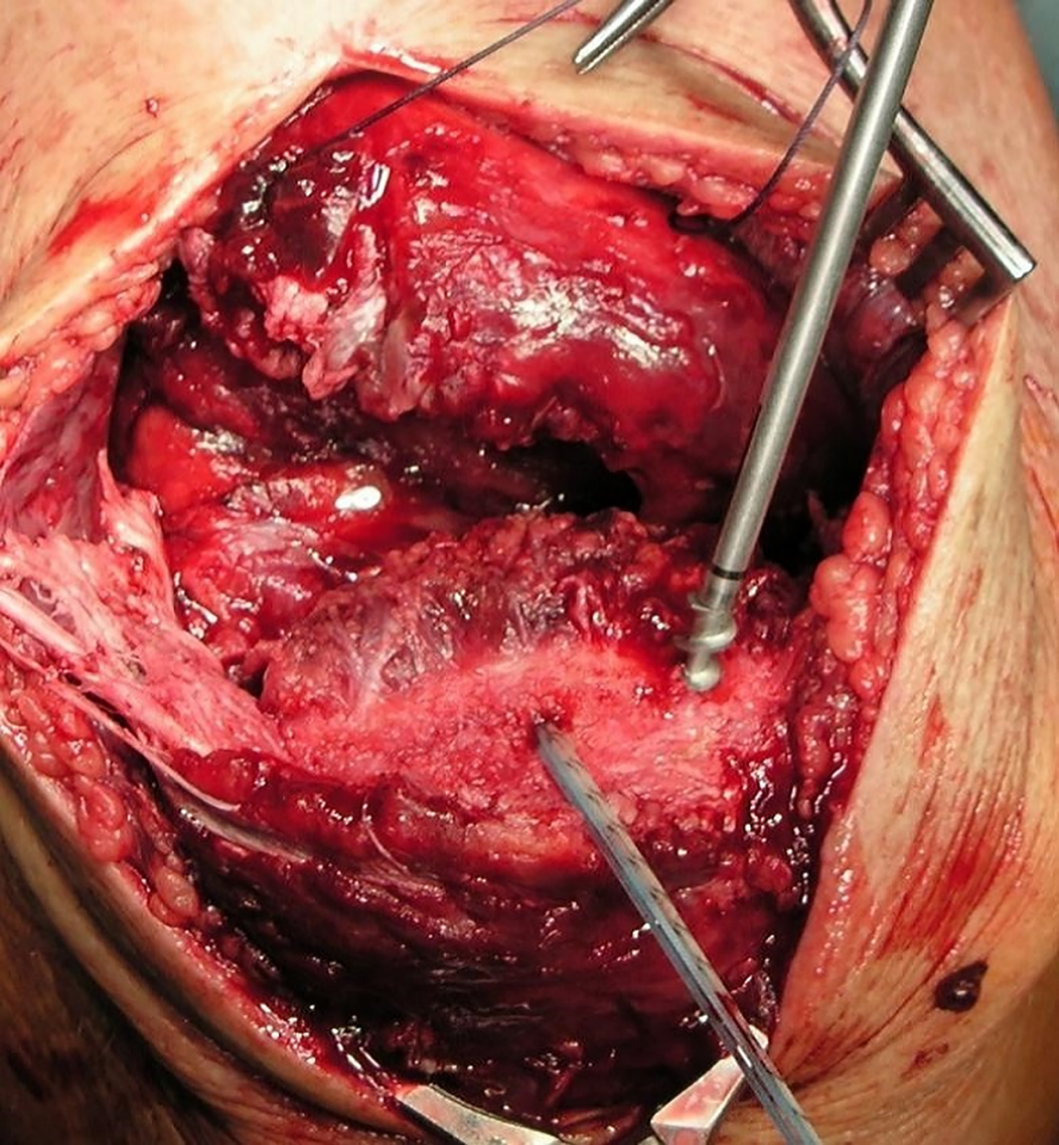
Placement of suture anchors.

SA repair required three pilot holes be created in the proximal pole of the patella with a 3.2-mm drill bit. Three 5.5-mm titanium corkscrew suture anchors armed with two strands of FiberWire and two strands of TigerWire (Arthrex Inc., Naples, FL) were then inserted into the holes. (Fig 2) The suture strands were then used to firmly grasp and pull the tendon towards the patella using a modified Mason-Allen stitch pattern. Patients were postoperatively placed in a ROM brace for 6 weeks. Initially locked in extension, the range of motion was postoperatively increased to 40° after 2 weeks and 60° after 4 weeks. Continuous passive motion (CPM) was performed with increasing flexion every 2 weeks (40°, 60°, and 90°). Partial weight-bearing was encouraged for the first two weeks after which full weight bearing was allowed as tolerated by the patient. (Table 1)

**Table 1:** SA group rehabilitation protocol.

Surgery ↓	2 weeks ↓	4 weeks ↓	6 weeks ↓	
ROM brace	Locked extension	40°	60°	Brace discontinued
Weight Bearing	Partial	Full as tolerated by patient		
CPM	40°	60°	90°	

ROM: range-of-motion, CPM: continuous passive motion

#### Outcomes

Follow-up consultations included physical and radiological examinations to determine standard scores (Lysholm and Tegner Scores [14], IKDC Score [15], VAS[16]), isokinetic quadriceps strength, as well as Insall-Salvati Index (ISI) [17]. Isokinetic testing was performed using a Biodex System 3 dynamometer (Biodex Medical Systems, Inc., Shirley, NY, USA). Quadriceps peak torque was measured throughout the range of motion of the knee at 60°/s and 240°/s. ‘Peak torque per body weight’ (PT/BW) was calculated for comparison between the two groups. Patients with concurrent bilateral ruptures were included in the analysis by using the mean PT/BW value of both legs, as previously done by Konrath et al [3]. Comparative measurements between involved and uninvolved leg were performed in 6 patients from the SA group and 5 from TS group. A difference of greater than 10% between legs is considered indicative of a deficit of muscle strength. In consonance with previous studies [3,18,19,20], a difference greater than 20% between the involved and uninvolved legs was considered significant. The ISI was determined by obtaining lateral radiographs of the affected and unaffected knee in 30° flexion.

### Statistical analysis

Descriptive statistics were calculated using SPSS (SPSS, Chicago, IL). Due to the small sample sizes, the Mann-Whitney *U* test for two independent samples was performed to compare the means of the two groups. All statistical tests were performed with 80% power and alpha = 0.05 for two sided tests, whereby p<0.05 was considered statistically significant.

## Results

**Fig 3:**
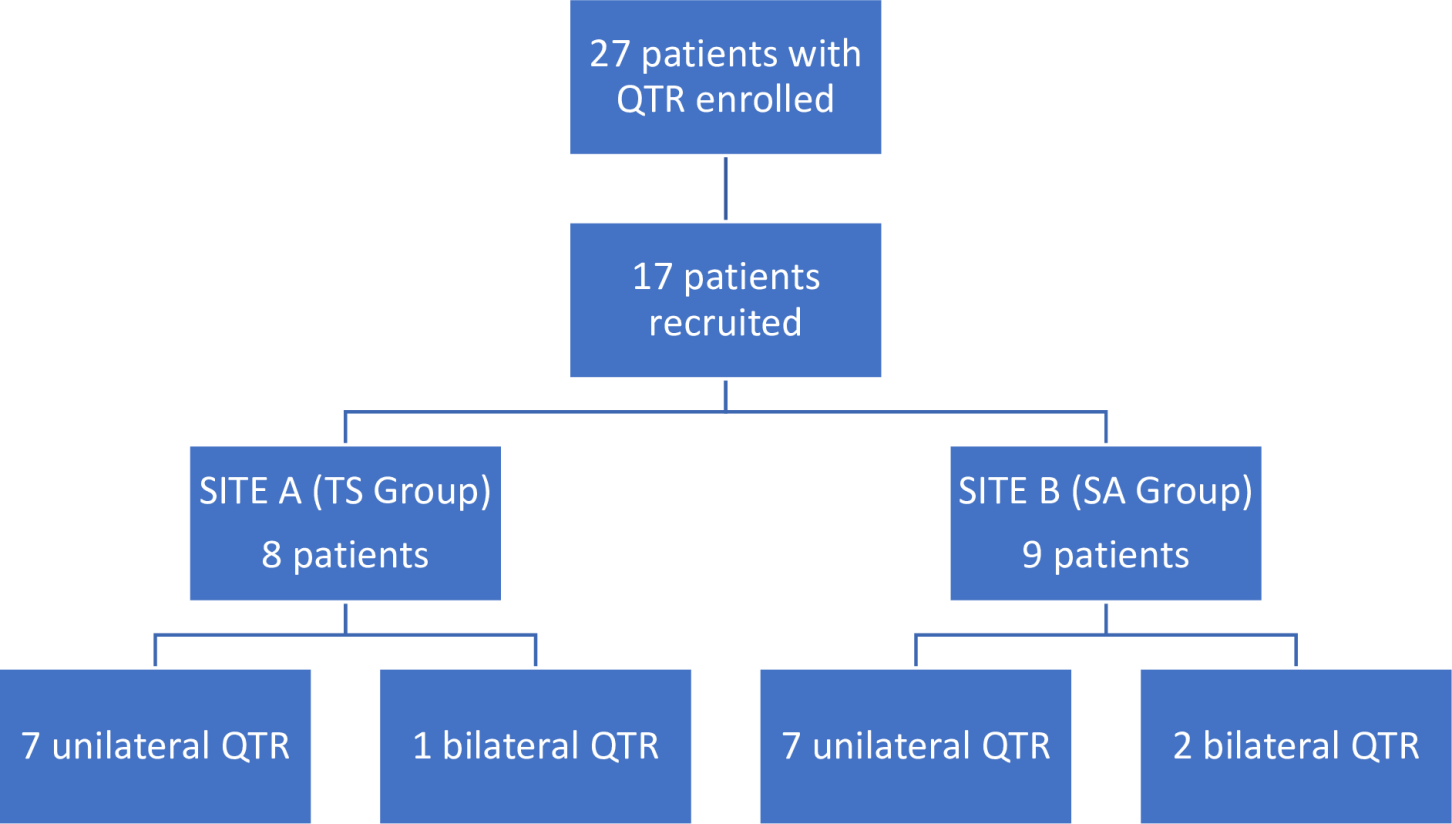
Recruitment protocol. SA: suture anchor, TS: transosseous suture, QTR: quadriceps tendon rupture.

Twenty-seven patients met the inclusion criteria of which 6 were lost to follow-up, 3 did not wish to participate in outcome evaluations, and 1 was unable to participate due to comorbidities. The 3 patients who declined to participate reported back by phone that they were satisfied with the results of the operation. The patient who could not participate because he was undergoing radiation treatment for lung cancer also reported good results. Of the 17 patients that were available for follow-up, 8 patients (1 patient with simultaneous bilateral rupture) had received TS repair and 9 patients (2 patients with simultaneous bilateral ruptures) SA repair (Fig 3).

Baseline characteristics were well-matched between the SA group and TS group (Table 2). The mean age of all patients was 60.4 years (SD: 10.7). 5 patients showed pre-disposing risk factors including diabetes, steroid abuse and statin use. 9 patients suffered QTR from indirect trauma. 7 patients fell down a flight of stairs, 7 patients slipped or tripped and fell, 1 fell while riding his bicycle, 1 had a motorcycle accident and 1 patient ruptured his quadriceps tendon playing football. 3 patients suffered simultaneous bilateral quadriceps tendon ruptures. No re-ruptures occurred in either group.

**Table 2:** Patient characteristics.

	SA group *n* = 9	TS group *n* = 8	*p* value
Age (years)	62.7 (SD: 8.8)	57.9 (SD: 12.7)	*n*.*s*.
Gender	9 men	8 men	*n*.*s*.
BMI (kg/m2)	29.1 (SD: 3.7)	29.3 (SD: 4.0)	*n*.*s*.
Follow-up (months)	46 (SD: 17)	29 (SD: 7)	*0*.*011*
Time-to-surgery (days)	4 (range: 0–14)	2 (range: 0–6)	*n*.*s*.

SA: suture anchor, TS: transosseous suture, BMI: body-mass index

### Scores

**Fig 4:**
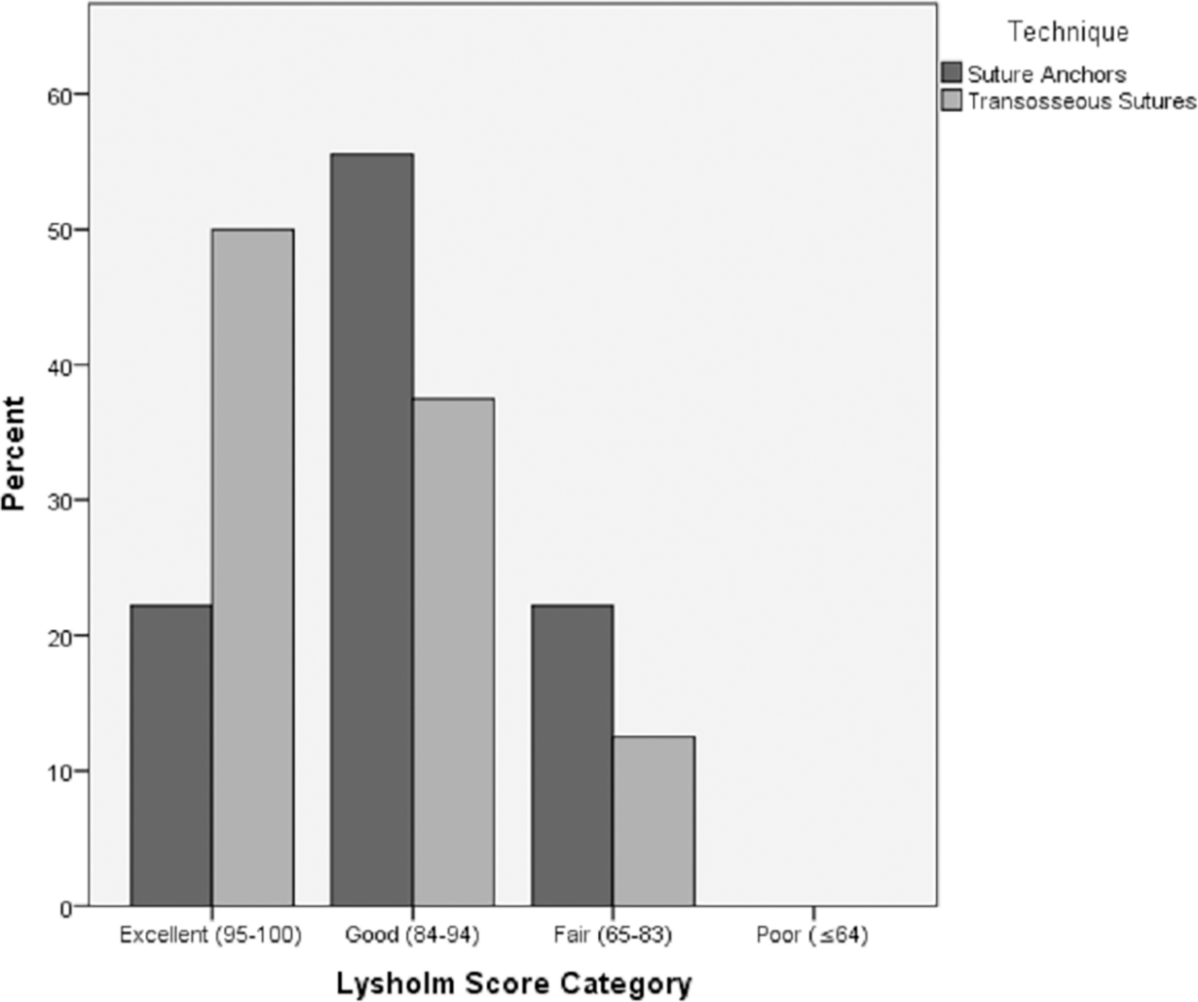
Distribution of Lysholm Score by group.

All patient scores are shown in Table 3. 7 out of 9 patients in the SA group, as well as 7 out of 8 patients in the TS group could be categorized as ‘good’ or ‘excellent’ according to the Lysholm Score. The distribution of Lysholm Score among the groups is shown in Fig 4.

**Table 3:** Scores.

	SA group *n* = 9	TS group *n* = 8	*p* value
Lysholm Score	88 (SD: 10)	94 (SD: 7)	*n*.*s*.
Tegner Activity Score	4 (range: 3–5)	5 (range: 3–7)	*n*.*s*.
IKDC Score	76.0 (SD: 13.9)	85.1 (SD: 7.1)	*n*.*s*.
Visual Analog Scale	5 (SD: 6)	0 (SD: 0)	*n*.*s*.

SA: suture anchor, TS: transosseous suture, IKDC: International Knee Documentation Committee

### Isokinetic strength testing

Results of isokinetic testing are shown in Table 4. 6 out of 9 patients in the SA group and 5 out of 8 patients in the TS group were suitable for comparative isokinetic strength analyses between the affected and unaffected knee. During 60°/s extension, 3 out of 6 SA group patients and 1 out of 5 TS group patients demonstrated a deficit greater than 20%. At 240°/s, such a deficit was observed in 4 out of 6 SA group patients and 0 out of 5 TS group patients.

**Table 4:** Isokinetic testing.

	SA group *n* = 9	TS group *n* = 8	*p* value
PT/BW at 60°/s (NM/kg)	122.5 (SD: 44.9)	158.9 (SD: 41.9)	0.200
PT/BW at 240°/s (NM/kg)	77.2 (SD: 31.1)	104.7 (SD: 30.4)	0.167
	*n* = 6	*n* = 5	
Deficit at 60°/s (%)	26.6 (SD: 27.8)	0.7 (SD: 15.7)	0.082
Deficit at 240°/s (%)	27.2 (SD: 23.5)	0.3 (SD: 15.7)	0.052

SA: suture anchor, TS: transosseous suture, PT/BW: peak torque per body weight, °/s degrees per second, NM/kg: newton-meters per kilogram

### Range of motion

The mean range of motion of all operated legs was 132° (120° to 146°) in the SA group and 138° (120° to 150°) in the TS group. One patient in the SA group had an extensor lag of 30°. All but one patient reached a range of motion within 10° of the uninvolved leg.

### Insall-Salvati Index

The mean Insall-Salvati Index of the operated legs in the SA group and TS group was 1.03 (0.76 to 1.21) and 0.98 (0.84 to 1.13), respectively. Where possible, radiographs of the contralateral leg were taken for comparison. The mean difference between the operated and healthy leg was 0.01 (-0.08 to 0.05) in the SA group and 0.04 (-0.06 to 0.18) in the TS group.

## Discussion

The most important finding of this study is, that patients who were treated on ruptures of the quadriceps tendon showed good to excellent outcomes, independent of the surgical technique used. Furthermore, this study showed no significant differences in outcomes between suture anchor repair and transosseous suture repair. To the author’s knowledge, this is the first study directly comparing clinical outcomes following suture anchor repair with clinical outcomes following transosseous suture repair.

The mean age of all patients at the time of injury was 60.4 years (35 to 73 years), which is comparable to other published patient populations. On average, patients from the SA group were almost 5 years (4.8 years) older than patients in the TS group. There were 17 male patients and no female patients. Patients from both groups were overweight with a mean body mass index (BMI) of 29.2 kg/m^2^.

The detrimental effects of delayed repair (> 3 weeks) of QTR are well documented in literature. Throughout this study, the mean time-to-surgery was 4 and 2 days in the SA and TS groups, respectively. With both participating hospitals being trauma centers and having the capacity to carry out the QTR repairs promptly, none of the surgeries were classed as ‘late’ (>3 weeks). The longest time-to-surgery was 2 weeks due to unclear ultrasound findings requiring follow-up using magnetic resonance imaging (MRI).

Mean Lysholm Scores [14] achieved by both groups were comparable to previously published results. [6,9,20,21] Although a difference of 6 points in mean Lysholm Score between the groups (SA: 88, TS: 94) was recorded, statistical analysis revealed this was not significant. The mean Tegner Activity Score of the TS group was slightly higher than that of the SA group (5 vs. 4). The increased level of activity among patients in the TS group might be explained by the 4.8-year difference in mean age. Mean IKDC Scores were comparable or higher than in literature. [22] Four patients in the SA group, compared to none in the TS group, indicated mild residual pain on the VAS. The cause of this difference, potentially attributable to the suture anchors remaining inside the patella of the SA patients, remains uncertain.

Principally, published studies evaluate quadriceps strength by conducting isokinetic tests on both the healthy and affected leg to determine any deficits. In order to compare all participating QTR patients, this study used the PT/BW of the operated leg or the mean PT/BW of both knees in bilateral injuries. The results of this analysis were comparable to results published by Konrath et al [3], where patients achieved a mean PT/BW of 116.57% at 60°/s and 68.75% at 240°/s. The TS group showed greater PT/BW than the SA group at both 60°/s (TS: 158.9%, SA: 122.5%) and 240°/s (TS: 104.7%, SA: 77.2%). However, statistical analysis of mean PT/BW at 60°/s identified no significant differences between the two groups.

In a subgroup analysis of patients that had unilateral QTR along with no prior injury to the contralateral leg (SA: n = 6, TS: n = 5), the quadriceps strength of the operated leg was compared to that of the healthy leg. At 60°/s, 3 of 6 patients in the SA group and 1 of 5 patients in the TS group showed deficit of greater than 20%. At 240°/s, 4 of 6 patients in the SA group, and 0 of 5 patients in the TS group showed a deficit greater than 20%. This contrasts Konrath et al [3] where more than half of their unilateral QTR patients had significant (> 20%) quadriceps strength deficits at 60°/s at a mean follow-up of 4 years. Konrath et al [3] were not able to discern the reason for the high number of patients with significant residual strength deficits. Although they allowed for early mobilization and immediate partial weight bearing, their patients did not perform as well as patients in other published series regarding isokinetic testing. Rougraff et al [19] reported 15% of patients with significant (>20%) strength deficit while Wenzl [21] et al reported 38% of patients with significant strength deficit at low speed.

All operated legs, in both groups, were evaluated using the ISI. All but two patients in the SA group registered normal mean values (SA group: 1.03, TS group: 0.98); one patient recorded 1.26 and the other 0.76. In comparison to the healthy contralateral leg, the recorded mean differences were minimal (SA = 0.01; TS = 0.04). These results are in concordance with those from Konrath et al [3], where the mean ISI value was 0.97, and the average difference was 0.04.

All patients reached a ROM greater than 120° in the operated leg with no significant differences between the means of the SA and TS group (SA = 132, TS = 138, p = n.s.). These results are consistent with those from previous studies. [6,9,20,21,23] All patients in the SA group, except one with an extensor lag of 30°, achieved a ROM within 10° of the uninjured leg.

Current literature suggests several benefits of SA repair over TS repair for treatment of QTR. These improvements include biomechanical superiority allowing more aggressive rehabilitation as well as intraoperative advantages such as reduced operative time, less tissue dissection, and no dissection of the patella tendon. Despite this growing body of evidence, TS repair has remained the gold-standard for repair of QTR. So far, only biomechanical studies, along with small case series, have been published focusing on the outcomes of SA repair. Without large randomized prospective studies it is hard for SA repair to become the predominant surgical technique used to repair QTR. The high cost of SA, far exceeding the cost of TS, further hinders this transition.

While many authors prefer a period of immobilization of at least six weeks after quadriceps tendon repair [18,21,23], recent studies have proposed earlier and more functional rehabilitation protocols. [20,22] Of the few published reports about the use of suture anchors for the repair of QTR, authors have advocated for early rehabilitation protocols. [5,7,8,9] Although several benefits of early rehabilitation have been described, Wenzl et al [21], as well as Langenhan et al [22] showed no significant differences between immobilization and early mobilization after repair of QTR. In this study, patients from the TS group followed a more conservative postoperative protocol—immobilization in a cast for an average of 7.5 weeks—whereas patients in the SA group followed a postoperative protocol involving a ROM brace and allowing early passive motion in a CPM device. Furthermore, the aggressive SA rehabilitation protocol encouraged for full weight bearing with increasing range of motion from two weeks post-surgery. The ROM brace in the SA group was discontinued after six weeks and free ambulation was enforced.

Although no statistically significant difference in clinical outcomes was shown at a mean follow-up of 46 and 29 months for the SA and TS groups, respectively, the disadvantages to cast immobilization, as argued by West et al [20], cannot be ruled out. Casting imposes a number of difficulties on patients, especially in cases of simultaneous bilateral ruptures. Such patients have trouble moving due to the weight of the cast; even simple actions such as getting in and out of cars, as well as sitting in cars can prove very difficult for them. Hence, casting often requires lengthy hospital stays and placement in nursing homes until the cast is removed. Replacing casts with ROM braces may avoid these issues and allow the patient to return home sooner. Whether this leads to quantifiable outcome differences early in the rehabilitation process remains unknown. Additional prospective studies are needed to further investigate outcomes at earlier points in the rehabilitation process.

The biomechanical superiority of SA allowing for earlier rehabilitation, as well as the mentioned intraoperative benefits may lead surgeons to favor SA repair over TS repair. Allowing for early mobilization with a ROM brace may prove especially beneficial in patients with simultaneous bilateral quadriceps tendon ruptures.

Several limitations of the study can be discerned, most importantly the retrospective as well as the two-center design. The participation of multiple surgeons resulted in slight differences in surgical techniques (eg different choice of suture material or suture pattern). Furthermore, the scarcity of quadriceps tendon ruptures also limited the sample size. The lack of exclusion criteria in terms of age and activity level, leading to an inhomogeneous patient population, along with 17 out of 27 patients (63%) participating in follow-up examinations might bias our results. Due to advanced age and the relatively long time required for the follow-up examination, for the time being no higher number of patients could be included in the study. Further studies that cover this topic more closely are being carried out at present and will therefore provide more information in the near future.

## Conclusion

Patients who underwent suture anchor repair for quadriceps tendon rupture and followed an aggressive early rehabilitation protocol showed outcomes as good as patients who were treated with transosseous sutures and followed a conservative rehabilitation protocol. Although patients from the transosseous suture group showed slightly better results in Lysholm Score, IKDC Score and quadriceps strength testing, these differences were not statistically significant. From a clinical perspective, suture anchors present a valid alternative to the gold-standard of transosseous sutures with added benefits early in the rehabilitation process.

## Supporting information

S1 Table Data set. (XLSX)

## Abbreviations

BMI: body mass index
CPM: continuous passive motion
IKDC: International Knee Documentation Committee
ISI: Insall-Salvati Index
MRI: magnetic resonance imaging
PDS: polydioxane sutures
PT: peak torque
PT/BW: peak torque per body weight
QTR: Quadriceps tendon rupture
SA: suture anchor
TS: transosseous suture
VAS: visual analog scale
